# Methidathion: *S*-(5-meth­oxy-2-oxo-2,3-dihydro-1,3,4-thia­diazol-3-yl)methyl *O*,*O*-dimethyl phospho­rodithio­ate

**DOI:** 10.1107/S1600536811005241

**Published:** 2011-02-19

**Authors:** Hyunjee Kim, Yong Woon Shin, Ki-Min Park, Jineun Kim, Tae Ho Kim

**Affiliations:** a Department of Chemistry and Research Institute of Natural Sciences, Gyeongsang National University, Jinju 660-701, Republic of Korea; b Test & Analytical Laboratory, Korea Food & Drug Administration, 123-7 Yongdang-dong, Busan 608-829, Republic of Korea

## Abstract

The title compound, C_6_H_11_N_2_O_4_PS_3_, crystallizes with two independent mol­ecules in the asymmetric unit. The dihedral angles between the thia­diazole ring planes and the PS_2_ planes of the phospho­rodithio­ate group are 86.51 (5) and 56.33 (5)° in the two mol­ecules. In the crystal, weak inter­molecular S⋯S [3.570 (8) Å] inter­actions and C—H⋯O and C—H⋯N hydrogen bonds contribute to the stabilization of the packing.

## Related literature

For the toxicity and insecticidal activity of the title compound, see: Altuntas *et al.* (2002 ▶). For related structures, see: Rohrbaugh *et al.* (1976 ▶).
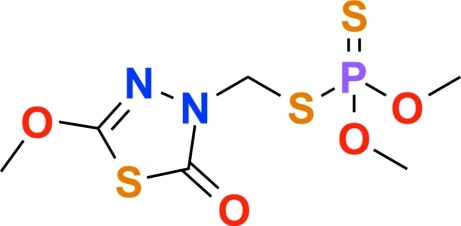


## Experimental

### 

#### Crystal data

C_6_H_11_N_2_O_4_PS_3_
*M* *_r_* = 302.32
Monoclinic, 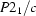

*a* = 12.3944 (2) Å
*b* = 10.8056 (1) Å
*c* = 19.3631 (3) Å
β = 102.815 (1)°
*V* = 2528.68 (6) Å^3^
*Z* = 8
Mo *K*α radiation
μ = 0.71 mm^−1^
*T* = 173 K
0.30 × 0.27 × 0.19 mm

#### Data collection

Bruker APEXII CCD diffractometer
Absorption correction: multi-scan (*SADABS*; Sheldrick, 1996 ▶) *T*_min_ = 0.815, *T*_max_ = 0.877
20918 measured reflections
5513 independent reflections
4682 reflections with *I* > 2σ(*I*)
*R*_int_ = 0.024

#### Refinement

*R*[*F* ^2^ > 2σ(*F* ^2^)] = 0.033
*wR*(*F* ^2^) = 0.089
*S* = 1.07
5513 reflections
289 parameters
H-atom parameters constrained
Δρ_max_ = 0.98 e Å^−3^
Δρ_min_ = −0.55 e Å^−3^

### 

Data collection: *APEX2* (Bruker, 2006 ▶); cell refinement: *SAINT* (Bruker, 2006 ▶); data reduction: *SAINT*; program(s) used to solve structure: *SHELXTL* (Sheldrick, 2008 ▶); program(s) used to refine structure: *SHELXTL*; molecular graphics: *SHELXTL* and *DIAMOND* (Brandenburg, 1998 ▶); software used to prepare material for publication: *SHELXTL*.

## Supplementary Material

Crystal structure: contains datablocks global, I. DOI: 10.1107/S1600536811005241/jh2268sup1.cif

Structure factors: contains datablocks I. DOI: 10.1107/S1600536811005241/jh2268Isup2.hkl

Additional supplementary materials:  crystallographic information; 3D view; checkCIF report

## Figures and Tables

**Table 1:** Hydrogen-bond geometry (Å, °)

*D*—H⋯*A*	*D*—H	H⋯*A*	*D*⋯*A*	*D*—H⋯*A*
C1—H1*A*⋯O7^i^	0.98	2.53	3.464 (3)	159
C1—H1*B*⋯O5^ii^	0.98	2.53	3.484 (3)	164
C2—H2*B*⋯O6^iii^	0.98	2.59	3.373 (3)	138
C3—H3*A*⋯N2^i^	0.99	2.56	3.506 (3)	161
C9—H9*B*⋯O3	0.99	2.42	3.246 (2)	140

Symmetry codes: (i) 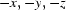; (ii) 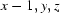; (iii) 
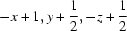
.

**Fig. 1.**
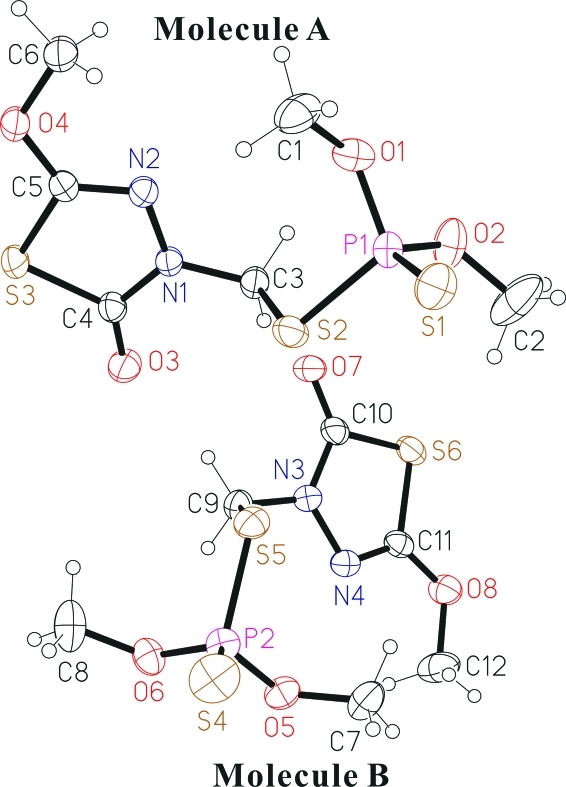
The molecular structure of the title compound with the atom numbering scheme. Displacement ellipsoids are drawn at the 50% probability level. H atoms are presented as a small spheres of arbitrary radius.

**Fig. 2.**
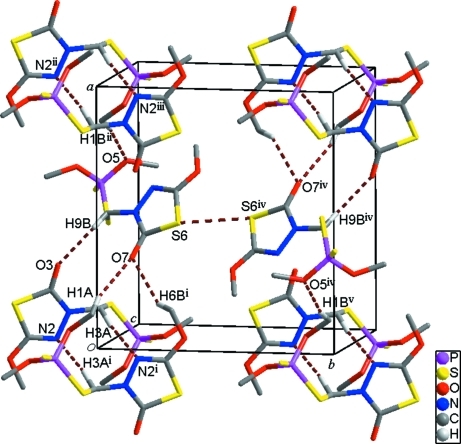
Crystal packing of the title compound with intermolecular C—H···O, C—H···N hydrogen bonds and S···S interactions shown as dashed lines. H atoms not involved in intermolecular interactions have been omitted for clarity. [Symmetry codes: (i) -x, -y, -z; (ii) x + 1, y, z; (iii) -x + 1, -y, -z; (iv) -x + 1, -y + 1, -z; (v) -x, -y + 1, -z.]

## supplementary crystallographic information

### Comment

Methidathion (systematic name: *S*-2,3-dihydro-5-methoxy-2-oxo-1,3,4- thiadiazol-3-ylmethyl *O*,*O*-dimethyl phosphorodithioate), is one of the most widely used organophosphate insecticides in agriculture and public health programmes (Altuntas *et al.* 2002). However it's crystal structure has not been reported yet.

In the title compound (Scheme 1, Fig. 1), crystallizes with two independent molecules in the asymmetric unit. The dihedral angles between the thiadiazol ring planes and the PS_2_ planes of the phosphorodithioate group are 86.51 (5)° and 56.33 (5)° in the two molecules. All bond lengths and bond angles are normal and comparable to those observed in similar structures (Rohrbaugh *et al.* 1976).

In the crystal structure, as shown in Fig. 2, weak intermolecular C—H···O, C—H···N hydrogen bonds (Table 1) and S···S interactions with 3.3372 (8) Å are observed (Table 1). These intermolecular interactions may be contribute to the stabilization of the packing.

### Experimental

The title compound was purchased from the Dr. Ehrenstorfer GmbH Company. Slow evaporation of a solution in CH_2_Cl_2_ gave single crystals suitable for X-ray analysis.

### Refinement

All H-atoms were positioned geometrically and refined using a riding model with d(C—H) = 0.99 Å, *U*_iso_ = 1.2*U*_eq_(C) for CH_2_ and d(C—H) = 0.98 Å, *U*_iso_ = 1.5*U*_eq_(C) for CH_3_ groups.

### Crystal data

**Table:**

C_6_H_11_N_2_O_4_PS_3_	*F*(000) = 1248
*M**_r_* = 302.32	*D*_x_ = 1.588 Mg m^−^^3^
Monoclinic, *P*2_1_/*c*	Mo *K*α radiation, λ = 0.71073 Å
Hall symbol: -P 2ybc	Cell parameters from 9930 reflections
*a* = 12.3944 (2) Å	θ = 2.5–28.2°
*b* = 10.8056 (1) Å	µ = 0.71 mm^−^^1^
*c* = 19.3631 (3) Å	*T* = 173 K
β = 102.815 (1)°	Block, colourless
*V* = 2528.68 (6) Å^3^	0.30 × 0.27 × 0.19 mm
*Z* = 8	

### Data collection

**Table:**

Bruker APEXII CCD diffractometer	5513 independent reflections
Radiation source: fine-focus sealed tube	4682 reflections with *I* > 2σ(*I*)
graphite	*R*_int_ = 0.024
φ and ω scans	θ_max_ = 27.0°, θ_min_ = 1.7°
Absorption correction: multi-scan (*SADABS*; Sheldrick, 1996)	*h* = −15→11
*T*_min_ = 0.815, *T*_max_ = 0.877	*k* = −11→13
20918 measured reflections	*l* = −24→24

### Refinement

**Table:**

Refinement on *F*^2^	Primary atom site location: structure-invariant direct methods
Least-squares matrix: full	Secondary atom site location: difference Fourier map
*R*[*F*^2^ > 2σ(*F*^2^)] = 0.033	Hydrogen site location: inferred from neighbouring sites
*wR*(*F*^2^) = 0.089	H-atom parameters constrained
*S* = 1.07	*w* = 1/[σ^2^(*F*_o_^2^) + (0.0424*P*)^2^ + 1.2139*P*] where *P* = (*F*_o_^2^ + 2*F*_c_^2^)/3
5513 reflections	(Δ/σ)_max_ = 0.001
289 parameters	Δρ_max_ = 0.98 e Å^−^^3^
0 restraints	Δρ_min_ = −0.55 e Å^−^^3^

### Special details

**Table:**

Geometry. All e.s.d.'s (except the e.s.d. in the dihedral angle between two l.s. planes) are estimated using the full covariance matrix. The cell e.s.d.'s are taken into account individually in the estimation of e.s.d.'s in distances, angles and torsion angles; correlations between e.s.d.'s in cell parameters are only used when they are defined by crystal symmetry. An approximate (isotropic) treatment of cell e.s.d.'s is used for estimating e.s.d.'s involving l.s. planes.
Refinement. Refinement of *F*^2^ against ALL reflections. The weighted *R*-factor *wR* and goodness of fit *S* are based on *F*^2^, conventional *R*-factors *R* are based on *F*, with *F* set to zero for negative *F*^2^. The threshold expression of *F*^2^ > σ(*F*^2^) is used only for calculating *R*-factors(gt) *etc*. and is not relevant to the choice of reflections for refinement. *R*-factors based on *F*^2^ are statistically about twice as large as those based on *F*, and *R*- factors based on ALL data will be even larger.

### Fractional atomic coordinates and isotropic or equivalent isotropic displacement parameters (Å^2^)

**Table:**

	*x*	*y*	*z*	*U*_iso_*/*U*_eq_	
P1	0.02509 (4)	0.14885 (4)	0.16800 (3)	0.02754 (12)	
P2	0.60901 (4)	−0.02370 (5)	0.25072 (3)	0.02917 (13)	
S1	0.01168 (5)	0.17732 (6)	0.26343 (3)	0.04165 (15)	
S2	0.15513 (4)	0.02823 (5)	0.16946 (3)	0.03550 (14)	
S3	0.16030 (4)	−0.35504 (4)	0.03186 (3)	0.03270 (13)	
S4	0.57301 (6)	−0.06480 (7)	0.33906 (3)	0.05122 (17)	
S5	0.46376 (4)	0.01769 (5)	0.17901 (3)	0.03259 (13)	
S6	0.48755 (4)	0.34626 (5)	−0.00393 (3)	0.03301 (13)	
O1	−0.07804 (11)	0.09769 (14)	0.11286 (7)	0.0355 (3)	
O2	0.04167 (15)	0.26370 (13)	0.12160 (8)	0.0459 (4)	
O3	0.31042 (11)	−0.17257 (13)	0.05006 (8)	0.0363 (3)	
O4	−0.05059 (11)	−0.37521 (12)	0.03211 (8)	0.0338 (3)	
O5	0.69519 (11)	0.08288 (13)	0.25149 (7)	0.0326 (3)	
O6	0.67189 (12)	−0.12180 (13)	0.21387 (8)	0.0347 (3)	
O7	0.35232 (12)	0.15068 (14)	−0.01300 (8)	0.0412 (4)	
O8	0.67746 (11)	0.41371 (13)	0.07718 (8)	0.0351 (3)	
N1	0.13193 (12)	−0.12835 (14)	0.05709 (8)	0.0243 (3)	
N2	0.02836 (13)	−0.18037 (14)	0.05340 (9)	0.0263 (3)	
N3	0.51734 (13)	0.14990 (15)	0.06949 (9)	0.0283 (4)	
N4	0.60999 (13)	0.22068 (15)	0.09789 (8)	0.0269 (3)	
C1	−0.14361 (19)	−0.0019 (2)	0.13290 (14)	0.0459 (6)	
H1A	−0.2042	−0.0227	0.0927	0.069*	
H1B	−0.1742	0.0246	0.1730	0.069*	
H1C	−0.0967	−0.0748	0.1465	0.069*	
C2	0.1050 (3)	0.3680 (3)	0.14990 (15)	0.0700 (9)	
H2A	0.1047	0.4285	0.1122	0.105*	
H2B	0.1813	0.3423	0.1703	0.105*	
H2C	0.0731	0.4055	0.1869	0.105*	
C3	0.14752 (16)	0.00052 (17)	0.07483 (10)	0.0273 (4)	
H3A	0.0853	0.0487	0.0464	0.033*	
H3B	0.2167	0.0298	0.0626	0.033*	
C4	0.21602 (15)	−0.20269 (17)	0.04761 (10)	0.0259 (4)	
C5	0.03364 (15)	−0.29610 (17)	0.04052 (10)	0.0263 (4)	
C6	−0.15623 (17)	−0.31980 (19)	0.03375 (14)	0.0383 (5)	
H6A	−0.2133	−0.3842	0.0272	0.057*	
H6B	−0.1755	−0.2586	−0.0043	0.057*	
H6C	−0.1515	−0.2791	0.0795	0.057*	
C7	0.6764 (2)	0.2031 (2)	0.27938 (12)	0.0426 (5)	
H7A	0.7376	0.2585	0.2760	0.064*	
H7B	0.6721	0.1949	0.3291	0.064*	
H7C	0.6069	0.2375	0.2520	0.064*	
C8	0.6314 (2)	−0.2470 (2)	0.20117 (15)	0.0536 (7)	
H8A	0.6811	−0.2940	0.1780	0.080*	
H8B	0.5570	−0.2456	0.1705	0.080*	
H8C	0.6287	−0.2864	0.2464	0.080*	
C9	0.50949 (17)	0.02671 (18)	0.09544 (10)	0.0291 (4)	
H9A	0.5829	−0.0133	0.1020	0.035*	
H9B	0.4572	−0.0210	0.0591	0.035*	
C10	0.43898 (16)	0.19916 (19)	0.01633 (11)	0.0305 (4)	
C11	0.60347 (16)	0.32287 (18)	0.06415 (10)	0.0275 (4)	
C12	0.7657 (2)	0.3958 (2)	0.13932 (13)	0.0494 (6)	
H12A	0.8163	0.4666	0.1448	0.074*	
H12B	0.8063	0.3199	0.1339	0.074*	
H12C	0.7342	0.3887	0.1813	0.074*	

### Atomic displacement parameters (Å^2^)

**Table:**

	*U*^11^	*U*^22^	*U*^33^	*U*^12^	*U*^13^	*U*^23^
P1	0.0317 (3)	0.0231 (2)	0.0290 (3)	0.00059 (19)	0.0094 (2)	−0.0009 (2)
P2	0.0291 (3)	0.0324 (3)	0.0282 (3)	−0.0032 (2)	0.0112 (2)	0.0027 (2)
S1	0.0511 (4)	0.0445 (3)	0.0318 (3)	−0.0008 (3)	0.0143 (3)	−0.0075 (2)
S2	0.0322 (3)	0.0394 (3)	0.0307 (3)	0.0102 (2)	−0.0021 (2)	−0.0081 (2)
S3	0.0272 (3)	0.0230 (2)	0.0480 (3)	0.00344 (19)	0.0086 (2)	−0.0059 (2)
S4	0.0610 (4)	0.0630 (4)	0.0361 (3)	−0.0113 (3)	0.0247 (3)	0.0075 (3)
S5	0.0229 (2)	0.0389 (3)	0.0378 (3)	−0.0022 (2)	0.0106 (2)	−0.0046 (2)
S6	0.0327 (3)	0.0342 (3)	0.0299 (3)	0.0100 (2)	0.0021 (2)	0.0020 (2)
O1	0.0268 (7)	0.0473 (9)	0.0317 (8)	0.0021 (6)	0.0051 (6)	0.0004 (7)
O2	0.0710 (11)	0.0257 (7)	0.0442 (9)	−0.0051 (7)	0.0200 (8)	0.0005 (7)
O3	0.0253 (7)	0.0347 (8)	0.0505 (9)	−0.0015 (6)	0.0119 (7)	−0.0092 (7)
O4	0.0269 (7)	0.0220 (6)	0.0529 (9)	−0.0008 (5)	0.0098 (7)	−0.0015 (6)
O5	0.0290 (7)	0.0361 (8)	0.0335 (8)	−0.0074 (6)	0.0089 (6)	−0.0008 (6)
O6	0.0360 (8)	0.0310 (7)	0.0394 (8)	0.0035 (6)	0.0131 (7)	0.0057 (6)
O7	0.0283 (8)	0.0419 (9)	0.0463 (9)	0.0062 (7)	−0.0068 (7)	−0.0131 (7)
O8	0.0297 (7)	0.0351 (8)	0.0396 (8)	−0.0035 (6)	0.0054 (6)	0.0071 (7)
N1	0.0219 (8)	0.0211 (7)	0.0304 (8)	−0.0001 (6)	0.0067 (7)	−0.0015 (6)
N2	0.0240 (8)	0.0231 (8)	0.0321 (9)	−0.0004 (6)	0.0071 (7)	−0.0003 (7)
N3	0.0264 (8)	0.0274 (8)	0.0283 (9)	0.0007 (6)	0.0002 (7)	−0.0022 (7)
N4	0.0238 (8)	0.0301 (8)	0.0262 (8)	0.0000 (6)	0.0041 (7)	−0.0002 (7)
C1	0.0336 (12)	0.0538 (14)	0.0530 (15)	−0.0132 (10)	0.0157 (11)	−0.0132 (12)
C2	0.098 (2)	0.0519 (16)	0.0517 (16)	−0.0435 (16)	−0.0026 (16)	0.0031 (13)
C3	0.0284 (10)	0.0230 (9)	0.0306 (10)	−0.0004 (8)	0.0069 (8)	−0.0014 (8)
C4	0.0275 (10)	0.0241 (9)	0.0261 (9)	0.0023 (7)	0.0057 (8)	−0.0020 (8)
C5	0.0256 (10)	0.0239 (9)	0.0292 (10)	0.0028 (7)	0.0056 (8)	0.0007 (8)
C6	0.0264 (11)	0.0295 (10)	0.0604 (15)	−0.0019 (8)	0.0127 (10)	−0.0041 (10)
C7	0.0519 (14)	0.0384 (12)	0.0360 (12)	−0.0105 (10)	0.0069 (11)	−0.0075 (10)
C8	0.0713 (18)	0.0287 (12)	0.0661 (17)	−0.0002 (11)	0.0269 (15)	0.0012 (11)
C9	0.0292 (10)	0.0279 (10)	0.0300 (10)	0.0007 (8)	0.0065 (8)	−0.0053 (8)
C10	0.0274 (10)	0.0327 (10)	0.0299 (10)	0.0088 (8)	0.0031 (9)	−0.0074 (8)
C11	0.0240 (9)	0.0324 (10)	0.0263 (10)	0.0050 (8)	0.0063 (8)	0.0003 (8)
C12	0.0428 (14)	0.0520 (14)	0.0460 (14)	−0.0172 (11)	−0.0056 (11)	0.0082 (12)

### Geometric parameters (Å, °)

**Table:**

P1—O2	1.5720 (15)	N2—C5	1.280 (2)
P1—O1	1.5730 (15)	N3—C10	1.357 (3)
P1—S1	1.9166 (7)	N3—N4	1.388 (2)
P1—S2	2.0681 (7)	N3—C9	1.434 (2)
P2—O5	1.5685 (14)	N4—C11	1.277 (2)
P2—O6	1.5770 (15)	C1—H1A	0.9800
P2—S4	1.9135 (7)	C1—H1B	0.9800
P2—S5	2.0640 (8)	C1—H1C	0.9800
S2—C3	1.838 (2)	C2—H2A	0.9800
S3—C5	1.7363 (19)	C2—H2B	0.9800
S3—C4	1.7852 (19)	C2—H2C	0.9800
S5—C9	1.832 (2)	C3—H3A	0.9900
S6—C11	1.739 (2)	C3—H3B	0.9900
S6—C10	1.774 (2)	C6—H6A	0.9800
O1—C1	1.452 (3)	C6—H6B	0.9800
O2—C2	1.413 (3)	C6—H6C	0.9800
O3—C4	1.205 (2)	C7—H7A	0.9800
O4—C5	1.331 (2)	C7—H7B	0.9800
O4—C6	1.446 (2)	C7—H7C	0.9800
O5—C7	1.446 (3)	C8—H8A	0.9800
O6—C8	1.445 (3)	C8—H8B	0.9800
O7—C10	1.217 (2)	C8—H8C	0.9800
O8—C11	1.329 (2)	C9—H9A	0.9900
O8—C12	1.448 (3)	C9—H9B	0.9900
N1—C4	1.360 (2)	C12—H12A	0.9800
N1—N2	1.389 (2)	C12—H12B	0.9800
N1—C3	1.437 (2)	C12—H12C	0.9800
			
O2—P1—O1	94.54 (9)	N1—C3—H3B	109.3
O2—P1—S1	118.33 (6)	S2—C3—H3B	109.3
O1—P1—S1	118.66 (6)	H3A—C3—H3B	107.9
O2—P1—S2	107.77 (7)	O3—C4—N1	126.98 (18)
O1—P1—S2	107.54 (6)	O3—C4—S3	126.24 (15)
S1—P1—S2	108.78 (3)	N1—C4—S3	106.78 (13)
O5—P2—O6	95.40 (8)	N2—C5—O4	125.36 (17)
O5—P2—S4	117.08 (6)	N2—C5—S3	117.62 (15)
O6—P2—S4	119.02 (6)	O4—C5—S3	117.00 (13)
O5—P2—S5	109.75 (6)	O4—C6—H6A	109.5
O6—P2—S5	106.63 (6)	O4—C6—H6B	109.5
S4—P2—S5	108.07 (3)	H6A—C6—H6B	109.5
C3—S2—P1	102.80 (7)	O4—C6—H6C	109.5
C5—S3—C4	88.23 (9)	H6A—C6—H6C	109.5
C9—S5—P2	102.19 (7)	H6B—C6—H6C	109.5
C11—S6—C10	88.05 (9)	O5—C7—H7A	109.5
C1—O1—P1	119.85 (14)	O5—C7—H7B	109.5
C2—O2—P1	122.67 (16)	H7A—C7—H7B	109.5
C5—O4—C6	114.88 (15)	O5—C7—H7C	109.5
C7—O5—P2	119.87 (13)	H7A—C7—H7C	109.5
C8—O6—P2	121.24 (14)	H7B—C7—H7C	109.5
C11—O8—C12	114.92 (16)	O6—C8—H8A	109.5
C4—N1—N2	118.77 (15)	O6—C8—H8B	109.5
C4—N1—C3	122.56 (16)	H8A—C8—H8B	109.5
N2—N1—C3	118.57 (14)	O6—C8—H8C	109.5
C5—N2—N1	108.60 (15)	H8A—C8—H8C	109.5
C10—N3—N4	118.32 (16)	H8B—C8—H8C	109.5
C10—N3—C9	122.48 (17)	N3—C9—S5	114.67 (13)
N4—N3—C9	119.16 (16)	N3—C9—H9A	108.6
C11—N4—N3	108.72 (16)	S5—C9—H9A	108.6
O1—C1—H1A	109.5	N3—C9—H9B	108.6
O1—C1—H1B	109.5	S5—C9—H9B	108.6
H1A—C1—H1B	109.5	H9A—C9—H9B	107.6
O1—C1—H1C	109.5	O7—C10—N3	126.5 (2)
H1A—C1—H1C	109.5	O7—C10—S6	126.18 (16)
H1B—C1—H1C	109.5	N3—C10—S6	107.35 (14)
O2—C2—H2A	109.5	N4—C11—O8	125.39 (18)
O2—C2—H2B	109.5	N4—C11—S6	117.48 (15)
H2A—C2—H2B	109.5	O8—C11—S6	117.12 (14)
O2—C2—H2C	109.5	O8—C12—H12A	109.5
H2A—C2—H2C	109.5	O8—C12—H12B	109.5
H2B—C2—H2C	109.5	H12A—C12—H12B	109.5
N1—C3—S2	111.76 (13)	O8—C12—H12C	109.5
N1—C3—H3A	109.3	H12A—C12—H12C	109.5
S2—C3—H3A	109.3	H12B—C12—H12C	109.5
			
O2—P1—S2—C3	−55.63 (9)	C3—N1—C4—O3	−2.7 (3)
O1—P1—S2—C3	45.23 (9)	N2—N1—C4—S3	0.3 (2)
S1—P1—S2—C3	174.93 (7)	C3—N1—C4—S3	176.58 (14)
O5—P2—S5—C9	61.04 (9)	C5—S3—C4—O3	179.15 (19)
O6—P2—S5—C9	−41.17 (9)	C5—S3—C4—N1	−0.10 (14)
S4—P2—S5—C9	−170.21 (7)	N1—N2—C5—O4	−178.69 (17)
O2—P1—O1—C1	−169.51 (15)	N1—N2—C5—S3	0.2 (2)
S1—P1—O1—C1	−43.64 (17)	C6—O4—C5—N2	4.0 (3)
S2—P1—O1—C1	80.24 (15)	C6—O4—C5—S3	−174.91 (15)
O1—P1—O2—C2	162.2 (2)	C4—S3—C5—N2	−0.09 (17)
S1—P1—O2—C2	36.1 (2)	C4—S3—C5—O4	178.94 (16)
S2—P1—O2—C2	−87.7 (2)	C10—N3—C9—S5	−101.82 (19)
O6—P2—O5—C7	178.26 (15)	N4—N3—C9—S5	80.34 (19)
S4—P2—O5—C7	−55.19 (16)	P2—S5—C9—N3	−105.14 (14)
S5—P2—O5—C7	68.42 (15)	N4—N3—C10—O7	−178.22 (18)
O5—P2—O6—C8	177.37 (18)	C9—N3—C10—O7	3.9 (3)
S4—P2—O6—C8	52.25 (19)	N4—N3—C10—S6	3.0 (2)
S5—P2—O6—C8	−70.14 (18)	C9—N3—C10—S6	−174.84 (14)
C4—N1—N2—C5	−0.3 (2)	C11—S6—C10—O7	179.08 (19)
C3—N1—N2—C5	−176.80 (17)	C11—S6—C10—N3	−2.15 (14)
C10—N3—N4—C11	−2.3 (2)	N3—N4—C11—O8	179.59 (17)
C9—N3—N4—C11	175.65 (16)	N3—N4—C11—S6	0.3 (2)
C4—N1—C3—S2	−101.78 (18)	C12—O8—C11—N4	−6.0 (3)
N2—N1—C3—S2	74.52 (18)	C12—O8—C11—S6	173.21 (16)
P1—S2—C3—N1	−120.69 (12)	C10—S6—C11—N4	1.10 (16)
N2—N1—C4—O3	−178.97 (19)	C10—S6—C11—O8	−178.22 (15)

### Hydrogen-bond geometry (Å, °)

**Table:**

*D*—H···*A*	*D*—H	H···*A*	*D*···*A*	*D*—H···*A*
C1—H1A···O7^i^	0.98	2.53	3.464 (3)	159
C1—H1B···O5^ii^	0.98	2.53	3.484 (3)	164
C2—H2B···O6^iii^	0.98	2.59	3.373 (3)	138
C3—H3A···N2^i^	0.99	2.56	3.506 (3)	161
C6—H6B···O7^i^	0.98	2.57	2.996 (2)	106
C9—H9B···O3	0.99	2.42	3.246 (2)	140

Symmetry codes: (i) −*x*, −*y*, −*z*; (ii) *x*−1, *y*, *z*; (iii) −*x*+1, *y*+1/2, −*z*+1/2.
